# Peroxydisulfate Activation by Pyrolysis Products of Iron Grinding Sludge and Polyethylene Glycol for Methylene Blue Degradation: Mechanism and Performance

**DOI:** 10.3390/nano15201585

**Published:** 2025-10-17

**Authors:** De-Feng Kong, Hui-Lai Liu, Yi Han, Ting Shi, De-Jin Wang, Xing Chen

**Affiliations:** 1 School of Resources and Environmental Engineering, Hefei University of Technology, Hefei 230009, China; kdf_001.student@sina.com (D.-F.K.); 2024820023@hfut.edu.cn (H.-L.L.); 2 Anhui Haoyue Ecological Technology Co., Ltd., Hefei 230009, China; 3 College of Resources and Environment, Anqing Normal University, Anqing 246011, China; yihan@whu.edu.cn (Y.H.); 18324988735@163.com (T.S.)

**Keywords:** waste utilization, degradation, iron grinding sludge, polyethylene, peroxydisulfate

## Abstract

The pollution problem of iron grinding sludge (IS) and polyethylene glycol (PEG) threatens the ecosystem and human health. In this study, an iron-rich catalyst (ISPEG) was prepared by co-pyrolysis of grinding sludge and polyethylene glycol and used to activate peroxydisulfate (PDS) for degrading organic wastewater. In the ISPEG/PDS system, methylene blue (MB) was almost completely removed within 60 min with an apparent rate constant (Kobs) of 0.32 min^−1^ and a wide range of pH. The effects of IS doping ratio, pyrolysis temperature, catalyst injection, PDS concentration, co-existing ions, and pH on MB removal were investigated. The results showed that ISPEG/PDS had a high removal rate of various organics in the water column. The catalytic mechanism of the ISPEG/PDS system was explored by free radical quenching, electron paramagnetic resonance, and frontier orbital theory studies, in which the main active substance for degrading SDZ was SO_4_^•−^. Finally, the degradation pathways of MB in the ISPEG/PDS system were analyzed by LC-MS. These results indicate that the ISPEG/PDS system has the potential to treat organic wastewater under the concept of waste control waste.

## 1. Introduction

Peroxydisulfate (PDS)-based advanced oxidation processes (AOPs) garnered significant attention in water treatment due to their high redox potential, extended half-life, and broad pH range [1,2,3]. In PDS activation studies, most researchers utilized transition metal ions (Cu^2+^, Fe^2+^, Ce^2+^, etc.) to activate PDS and produce SO_4_^•−^ [4,5,6]. Among these, iron-based materials such as Fe^0^, Fe_3_O_4_, and FeOOH were extensively studied for their abundance, availability, and environmental friendliness [7]. For instance, Fe_3_O_4_ was one of the most common heterogeneous catalysts for activating PDS. Post-use, the catalyst could be conveniently separated by an external magnetic field, thereby facilitating repeated degradation [8]. However, the structure and physicochemical properties of materials prepared by different methods varied, resulting in significant differences in PDS activation [9]. Therefore, there was an urgent need to develop an economical, highly active, and easily recoverable iron-based catalyst.

Nowadays, the demand for the use of plastics is increasing substantially because of their low price, versatile uses and easy production [10]. Annually, over 240 million tons of PEG products were produced globally. Only about 6–26% of PEG was recycled, while approximately 21–42% ended up in landfills, and the remainder entered the environment through various mismanagement routes [11]. Alternative, more economical or efficient methods must be developed to deal with the rapid accumulation of plastic waste. Recently, pyrolysis of plastics, including polyethylene, has attracted the interest of many research groups and companies. Pyrolysis is a thermochemical process that converts polyethylene glycol into liquid fuels, waxes and gaseous products. An obvious disadvantage of pyrolysis is the high temperatures required to achieve the correct decomposition temperature. However, the high energy requirement can be overcome by the use of catalysts. Catalytic pyrolysis has several advantages over pyrolysis: (i) reduced energy requirements and operating costs due to lower temperatures required and (ii) improved selectivity of the desired products. Studies showed that by mixing Fe_3_O_4_ as an additive during pyrolysis, and using CO_2_ as the pyrolysis medium, PEG could be thermally converted into useful products [12]. The addition of Fe_3_O_4_ accelerated the thermal degradation of PE, and the collected char retained its adsorption capacity for dye molecules [13]. However, the cost of manually adding Fe_3_O_4_ was high. Therefore, to ensure a complete reduction of the iron precursor and adjust the pore structure, iron-containing chemicals and some activation chemicals were still needed [14]. To meet the growing demand for environmentally friendly processes, it was essential to minimize the consumption of chemicals and synthetic procedures as much as possible. Thus, co-pyrolysis of iron-rich waste materials with PEG might be a viable alternative.

Machining was an essential process in metal part production, but it generated a large amount of waste known as iron grinding sludge (IS) [15]. Iron grinding sludge, contaminated by abrasive particles from the grinding wheels used, contained a substantial amount of metal elements (Fe, Al, etc.), yet the accumulated iron-rich sludge still required final disposal, and the organic resources in the sludge were wasted [16]. When sent to the disposal enterprise for harmless treatment, grinding sludge needs to be contained within packaging containers that are constructed of Polyethylene glycol [17]. Traditional disposal methods such as landfill and incineration are not only costly but also pose risks of secondary pollution, constituting a significant threat to the environment. Although sludge-derived biochar has garnered attention in wastewater treatment, biochar obtained solely through pyrolysis of iron grinding sludge suffers from issues including an imbalanced Fe/C ratio, insufficient active sites, and limited electron transfer capacity, thereby restricting its application in water treatment [18]. Therefore, using IS as an Fe precursor and co-pyrolyzing it with PEG to form iron-rich biochar was feasible. This method resolves two critical issues inherent in conventional approaches: by synergistically optimising the pore structure and active sites through iron/carbon equilibrium, it achieves a significantly increased specific surface area compared to biochar produced solely by iron grinding sludge. Therefore, developing an efficient and sustainable technology for the resource utilisation of iron grinding sludge holds significant importance for environmental protection and sustainable development.

Here, we present the concept of “industrial waste solution for dye wastewater” using co-pyrolysis of iron mill sludge and polyethylene glycol for degradation of dye wastewater and report on a simple co-pyrolysis method that is applicable to the processing of waste polyethylene glycol into a solid catalyst for persulfate activation. This paper thoroughly investigated the phase changes in ISPEG at different temperatures. Methylene blue (MB) was chosen as the target pollutant since it was a typical organic wastewater contaminant. Furthermore, the study examined the impact of preparation conditions on catalyst performance, as well as the effects of PDS concentration, catalyst dosage, reaction temperature, and initial pH on MB catalytic degradation. Finally, the degradation mechanism and MB degradation pathways were analyzed.

## 2. Materials and Methods

### 2.1. Materials and Reagents

Iron grinding sludge (IS) and polyethylene glycol (PEG) were supplied by Anhui Haoyue Ecological Technology Co., Ltd (Hefei, China), Methanol (MeOH, 99.5%), tert-butanol (TBA, 99.9%), potassium persulfate (K_2_S_2_O_8_, ≥99.5%), p-benzoquinone (p-BQ, ≥99%), histidine (L-His, ≥99.5%), and 1,10-phenanthroline (C_12_H_8_N_2_, ≥97%) were all purchased from Macklin Biochemical Technology Co., Ltd. (Shanghai, China). Hydrochloric acid (HCl, ≥98%), sodium hydroxide (NaOH, ≥96%), methylene blue (MB), sodium bicarbonate (NaHCO_3_, ≥99.5%), sodium carbonate (Na_2_CO_3_, ≥99%), and sodium sulfate (Na_2_SO_4_, ≥99%) were all procured from Tianjin Chemical Reagent Research Institute Co., Ltd. (Tianjin, China). All chemicals were of analytical grade and used without further purification. Deionized water was used for all experiments.

### 2.2. Preparation of ISPEG

The iron sludge (IS) was dried in an oven at 60 °C until reaching constant weight, then ground into powder and sieved through a 200-mesh screen. Polyethylene glycol (PEG, 20 mg) was uniformly blended with varying proportions of IS (10%, 30%, 50%, 70%, 90%). The mixture was subsequently loaded into a tube furnace (GSL-1600X, shanghai, China) and heated from 20 °C to target temperatures (400–700 °C) at a heating rate of 10 °C/min under nitrogen atmosphere. The system was maintained at the target temperature for 30 min. The resulting product underwent centrifugation followed by three cycles of deionized water washing. After vacuum drying at 60 °C, the catalyst was obtained and designated as ISPEG-x, where x represents the mass percentage of IS (10–90%). The final catalyst powders were stored in desiccators for subsequent characterization and testing.

### 2.3. Method of Analysis

The chemical composition of IS and ISPEG was determined using X-ray fluorescence spectroscopy (XRF, ARL PERFORM’ X, Thermo Fisher Scientific, Waltham, MA, USA). The surface characteristics and morphology of the samples, as well as elemental mapping images, were analyzed using a scanning electron microscope (SEM, SU-8010, Hitachi High-Technologies Corporation, Tokyo, Japan). The elemental composition of the samples was examined with energy-dISPEGrsive X-ray spectroscopy (EDS, ESEM XL 30, FEI Company, Hillsboro, OR, USA). The crystalline phase of the samples was tested using an X-ray diffractometer (XRD, XRD-6100, Shimadzu Corporation, Tokyo, Japan). X-ray photoelectron spectroscopy (XPS) analysis, utilizing single crystal aluminum Kα X-rays (Thermo ESCALab 250XI, Thermo Fisher Scientific, Waltham, MA, USA), was conducted to study the chemical properties of ISPEG before and after the reaction. The magnetism of the catalyst was measured using a vibrating sample magnetometer (VSM, MPMS XL-7, Quantum Design, San Diego, CA, USA). For electron paramagnetic resonance (EPR, EMXplus-X, Bruker Corporation, Rheinstetten, Germany) analysis, 5,5-dimethyl-1-pyrroline-N-oxide (DMPO) was used as a non-selective spin trapping agent for SO_4_^•−^, •OH and O_2_^•−^ radicals. The concentration of iron ions was determined by UV-visible spectrophotometry (HACH) using the 1,10-phenanthroline method at a wavelength of 510 nm. Intermediate products in the MB degradation process were analyzed using liquid chromatography–mass spectrometry (Ultimate 3000 UHPLC-Q Exactive, Thermo Fisher Scientific, Waltham, MA, USA). Further testing details are provided in Text S1.

### 2.4. Experimental Procedures

All catalytic experiments were conducted in darkness at room temperature (25 °C) in a 150 mL beaker equipped with magnetic stirring (180 × 270 × 60 mm). This reactor contained 10 mg/L of MB and 1.2 mM of PDS. Typically, a certain amount of catalyst was added to the MB solution and allowed to react for 30 min to achieve adsorption equilibrium. Subsequently, a specific amount of PDS was introduced to initiate the experiment. At each time interval, samples were withdrawn from the reactor using a 5 mL syringe, filtered through a 0.22 μm filter, and then injected with an excess quenching agent to halt any remaining radical reactions. The concentration of MB was measured on a UV-vis spectrophotometer at 665 nm. The pH of the MB solution was adjusted using 0.1 M NaOH or HCl aqueous solutions. The reaction temperature was controlled via a water bath. After each degradation experiment, the catalyst was collected using an external magnetic field, washed several times with distilled water, and prepared for reuse testing.

Additionally, the effects of pyrolysis temperature (400, 500, 600, and 700 °C), catalyst dosage (0, 0.1, 0.2, 0.4, 0.6 g/L), PDS concentration (0, 0.4, 0.8, 1.2, and 1.6 mM), and pH (3, 5, 7, 9, 11) were investigated. The impact of CO_3_^2−^, SO_4_^2−^, and HNO_3_^−^ (10 mM) on MB degradation was also examined. After each experiment, the used catalyst was collected, washed with deionized water, and reused in the subsequent reaction cycle to explore the stability and reusability of ISPEG.

## 3. Results and Discussion

### 3.1. Characterization

**Figure 1:**
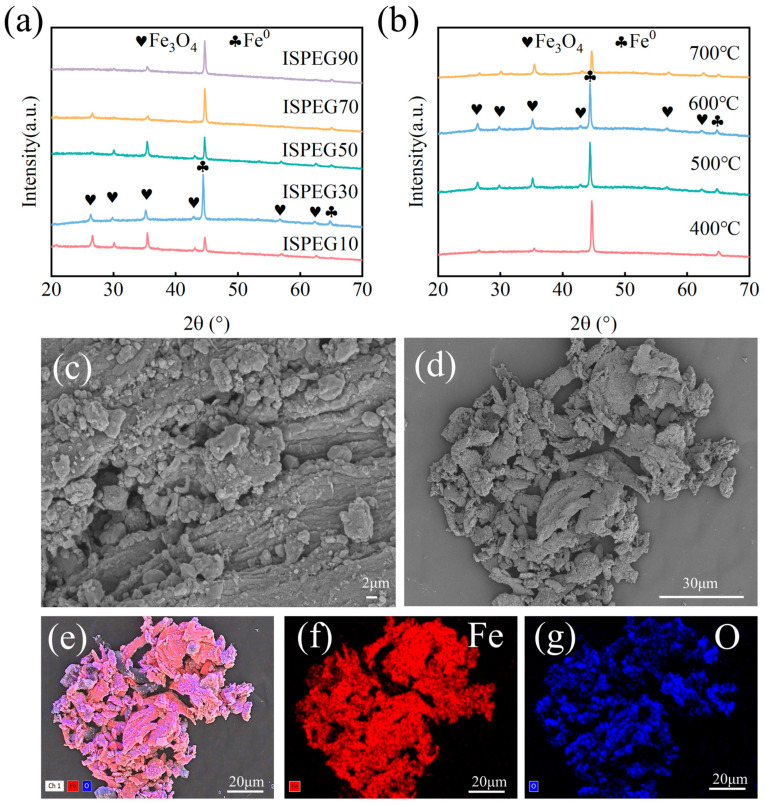
(**a**) XRD of different systems at 600 °C, (**b**) XRD of ISPEG30 at different temperatures, (**c**,**d**) SEM, (e) EDS, (**f**,**g**) elemental diagram of Fe, O.

**Figure 2:**
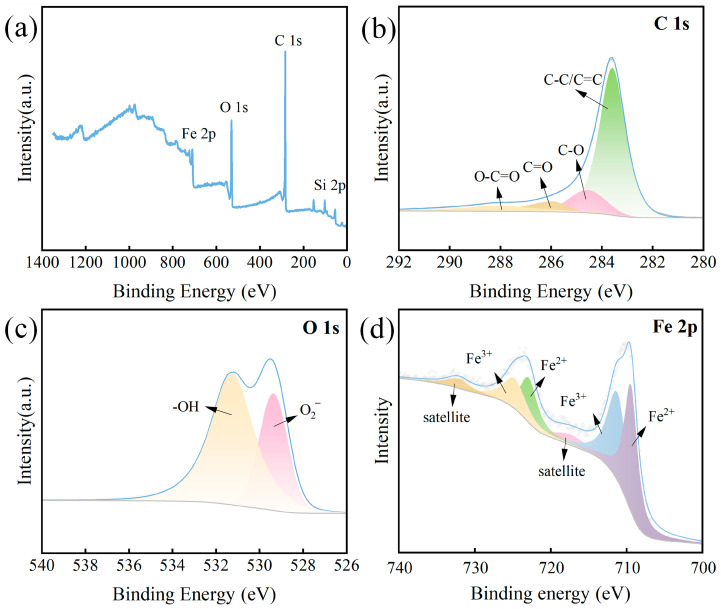
(**a**) XPS spectra of ISPEG30, (**b**) C 1s, (**c**) O 1s, (**d**) Fe 2p.

The XRD patterns of ISPEG at different proportions are shown in Figure 1a. As the IS content increases, the intensity of the Fe^0^ diffraction peaks increases, while the intensity of the Fe_3_O_4_ diffraction peaks correspondingly decreases. The characteristic peaks at 30.12°, 35.48°, 43.11°, 53.90°, 57.03° and 62.26° correspond to the (220), (311), (400), (422), (511), and (440) planes of Fe_3_O_4_ (JCPDS No. 01-19-0629) [19,20,21]. The typical XRD pattern of Fe^0^ exhibits peaks at 2θ values of 44.6°, 65.0°, and 82.3°, corresponding to the (110), (200), and (211) planes (JCPDS No. 01-087-0721), indicating the successful preparation of ISPEG [22,23]. The XRD patterns of ISPEG30 were examined at 400 °C, 500 °C, 600 °C, and 700 °C to further investigate the transformation of iron compounds during pyrolysis (Figure 1b). When the pyrolysis temperature increases from 400 °C to 600 °C, iron in ISPEG30 mainly exists in the form of Fe_3_O_4_ (JCPDS No. 01-19-0629), with higher signal peak intensities. Combining the degradation performance of the catalyst at different temperatures (Figure 2b), the degradation effect is optimal at 600 °C. In contrast, when the pyrolysis temperature rises to 700 °C, the Fe^0^ peak intensity weakens, leading to a decrease in the catalytic activity of Fe^0^.

The SEM image (Figure 1c,d) revealed that the surface of the ISPEG sample contains numerous irregular fine particles, potentially resulting from the aromatization and carbonization of hydrocarbons in the biochar plastic [24]. Additionally, Fe_3_O_4_ particles exhibited agglomeration due to magnetic effects, providing more active sites for interaction with wastewater. EDS observations (Figure 1e,f) confirmed the presence of Fe and O elements on the surface of the prepared ISPEG catalyst, with Fe uniformly distributed and accounting for approximately 67.05% of the atomic composition (Figure S1 in Supplementary Materials), further indicating the successful preparation of ISPEG, consistent with XRD results. The XRF analysis of IS and ISPEG was presented in Table S1, showing a higher metal content in ISPEG, with iron content at 95.6%. Figure S3a illustrates the magnetization curve of ISPEG, with a saturation magnetization of 168 emu·g^−1^. Consequently, under the influence of an external magnetic field, it can be effortlessly recovered from solids and liquids and applied in wastewater treatment.

The changes in functional groups on the surface of ISPEG30 were analyzed using XPS. The prepared ISPEG30 mainly consists of characteristic peaks of Fe 2p, Si 2p, C 1s, and O1s as shown in Figure 2a The XPS spectrum of C1s is shown in Figure 2b. The four functional groups of C-C/C=C (284.7 eV), C-O (285.7 eV), C=O (286.0 eV), and O-C=O (288.1 eV) were observed [25]. The spectrum of O1s was classified as the characteristic peaks of lattice oxygen in metal oxides (O_2_^−^) and surface hydroxyl groups (-OH) (Figure 2c) [4,26]. The two Fe 2p 3/2 peaks were located at 709.9 eV and 711.6 eV, corresponding to the less catalytically active Fe^3+^, and the two Fe 2p 1/2 were the characteristic peaks of Fe^2+^ at 708.3 eV and 708.4 eV, respectively (Figure 2d) [27,28,29]. These results further proved that ISPEG30 was successfully prepared.

As shown in the infrared spectrum (Figure S2 in Supplementary Materials), the peak at 3329 cm^−1^ corresponds to O-H bond vibration, while the peak at 2980 cm^−1^ corresponds to C-H bond stretching vibration [30,31]. The peak at 1051 cm^−1^ is attributed to C=O bond stretching vibration, and the band around 890 cm^−1^ corresponds to FeOOH bonds [32,33]. Notably, these peaks di-minish with increasing pyrolysis temperature, likely due to dehydration or deoxygenation reactions occurring during pyrolysis.

### 3.2. Degradation of MB by ISPEG

#### 3.2.1. Effects of Different Ratios

**Figure 3:**
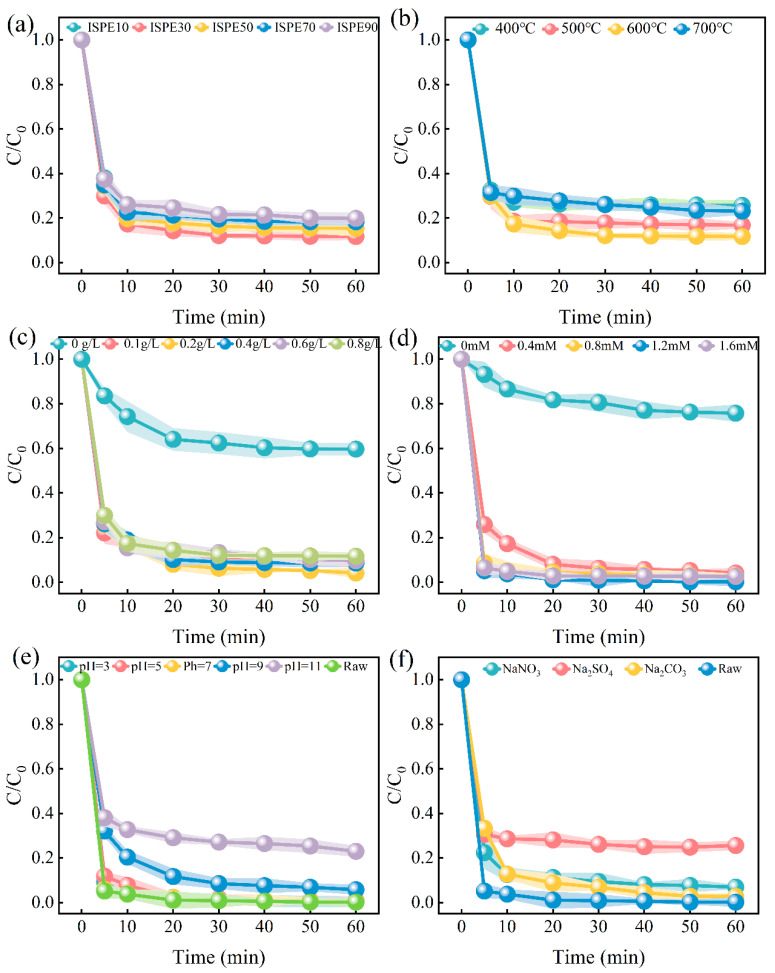
Effect of (**a**) different ratios of ISPEG, (**b**) pyrolysis temperatures, (**c**) ISPEG dosages, (**d**) PDS concentration, (**e**) pH, and (**f**) anions on MB degradation.

Figure 3 shows the single-factor experiments of ISPEG on MB degradation, and the parameters related to the kinetic analysis curves of each influencing factor are shown in Table S2 in Supplementary Materials. Figure 3a illustrates the impact of varying ISPEG proportions on MB degradation. Without the addition of IS, the removal rate of MB is merely 24.1%. In contrast, the removal rate significantly improves with ISPEG10, reaching 85.9% after 60 min. As the doping ratio of IS increases from 10% to 30%, the MB removal rate rises from 85.9% to 88.2%. This indicates that with the increase in IS content, more active sites are exposed, enhancing the catalytic activity of iron. However, excessive IS causes the samples to aggregate easily [34], reducing the catalyst’s specific surface area within the reaction system, thereby contributing minimally to the degradation of MB. Based on these results, we selected ISPEG30 for subsequent research.

#### 3.2.2. Effects of Different Pyrolysis Temperatures

The effect of different pyrolysis temperatures (400 °C, 500 °C, 600 °C, 700 °C) on MB degradation is shown in Figure 3b. With the increase in pyrolysis temperature, the removal rate of MB was 74.5%, 83.15, 84.65%, and 76.9%, indicating that the higher the temperature, the higher the catalytic activity of the prepared ISPEG for MB. XRD results (Figure 1b) showed that the increase in pyrolysis temperature led to changes in Fe_3_O_4_ crystals, which could provide more catalytic sites to promote the mass transfer of the target pollutant phase to the surface of the catalyst. However, higher temperatures lead to the sintering of the catalyst and a decrease in catalytic activity [35]. Therefore, a pyrolysis temperature of 600 °C was selected for all subsequent experiments.

#### 3.2.3. Effects of ISPEG Dosages

The influence of catalyst dosage on MB degradation is depicted in Figure 3c. As the ISPEG dosage increased to 0.2g/L, the degradation rate of MB after 60 min of reaction reached its peak. This is attributed to the higher catalyst dosage, which resulted in more active sites being generated [36]. However, when the catalyst dosage was increased from 0.2 g/L to 0.8 g/L, the MB degradation rate did not show a significant improvement. This can be explained by the possibility that the ${\mathrm{SO}}_{4}^{\cdot -}$ generated by the reaction of Fe^2+^ with PDS might be quenched (Equation (1)), releasing additional Fe^2+^, which induces a slower degradation process [37]. Therefore, a dosage of 0.2 g/L ISPEG was selected for subsequent experiments.

(1) $${\mathrm{Fe}}^{2+}+\;\mathrm{PDS}\;\to {\;\mathrm{Fe}}^{3+}+{\mathrm{SO}}_{4}^{\cdot -}$$

#### 3.2.4. Effects of PDS Concentration

The influence of ISPEG catalysts on the degradation of MB at varying PDS concentrations is depicted in Figure 3d. Pure ISPEG only achieved a 24.2% MB removal rate within 60 min, indicating that the catalyst’s adsorption was ineffective in removing MB. However, with a PDS concentration of 1.2 mM, 99.3% of MB was eliminated within the same period. When the PDS concentrations were 0.4 mM and 0.8 mM, the degradation of MB slightly decreased. This is attributed to the sulfate radicals derived from PDS; increasing the PDS concentration enhances radical production and accelerates the reaction rate, while excessively high concentrations may quench the sulfate radicals (Equations (2) and (3)) [38]. To ensure optimal economic efficiency, a PDS concentration of 1.2 mM was selected for subsequent experiments.

(2) $${\mathrm{SO}}_{4}^{\cdot -}+{\mathrm{SO}}_{4}^{\cdot -}\;\to {\;S}_{2}O_{8}^{2-}$$

(3) $${\mathrm{SO}}_{4}^{\cdot -}+S_{2}O_{8}^{2-}\;\to {\;S}_{2}O_{8}^{\cdot -}+{\;\mathrm{SO}}_{4}^{2-}$$

#### 3.2.5. Effects of pH

The initial pH plays a crucial role in the catalyst/PDS system. To explore the application scope of ISPEG, a broad pH range of 3–11 was studied (Figure 3e). The initial pH of the test solution was adjusted using HCl or NaOH. At initial pH values of 3, 5, 7, and 9, the removal efficiencies of MB were 99.5%, 99.2%, 98.9%, and 94.2%, respectively, indicating the optimal performance. It can be inferred that the ISPEG/PDS system can effectively degrade MB across a wide pH range, with the best performance at pH 3, which aligns with previous findings [39]. However, at pH 11, the MB removal rate dropped to 76.9%. Under acidic conditions, Fe^0^ undergoes corrosion, leading to a continuous release of Fe^2+^ (Equations (4) and (5)). Conversely, in alkaline solutions, electrostatic repulsion becomes more significant, hindering the interaction between the catalyst and PDS or MB [40]. Additionally, alkaline conditions facilitate the transformation of ${\mathrm{SO}}_{4}^{\cdot -}$ into the relatively less oxidative •OH (Equations (6) and (7)) [41,42]. Therefore, degradation efficiency is higher under acidic conditions.

(4) $$2{\mathrm{Fe}}^{0}+O_{2}+2H_{2}O\;\to \;{\mathrm{Fe}}^{2+}+4{\mathrm{OH}}^{-}$$

(5) $${\mathrm{Fe}}^{0}+S_{2}O_{8}^{2-}\;\to \;{\mathrm{Fe}}^{2+}+2{\mathrm{SO}}_{4}^{2-}$$

(6) $${\mathrm{SO}}_{4}^{\cdot -}+{\mathrm{OH}}^{-}\;\to \;\cdot \mathrm{OH}+{\mathrm{SO}}_{4}^{2-}$$

(7) $${\mathrm{SO}}_{4}^{\cdot -}+{\;H}_{2}O\;\to \;\cdot \mathrm{OH}+{\mathrm{HSO}}_{4}^{-}$$

#### 3.2.6. Effects of Anions

Inorganic anions, including HCO_3_^−^, CO_3_^2−^, and NO_3_^−^, are commonly present in most practical wastewater and adversely affect the activation of PDS. They can react with free radicals to form secondary radicals with weaker oxidizing capabilities. This experiment investigated the effects of three typical inorganic ions (HCO_3_^−^, CO_3_^2−^, and HNO_3_^−^) on the degradation of MB (Figure 3f). The inhibitory effect of anions on MB degradation follows the sequence HCO_3_^−^ > NO_3_^−^ > CO_3_^2−^. The results indicated that the presence of CO_3_^2−^ exhibits an inhibitory effect, with high concentrations of CO_3_^2−^ capable of quenching SO_4_^2−^• and •OH (Equations (8) and (9)). According to reports, NO_3_^−^ can act as a scavenger to consume SO_4_^2−^ and •OH radicals. Figure 3f could be explained that NO_3_^−^ generates NO_3_^•−^ with a lower redox potential (Equation (10)). HCO_3_^−^ can capture SO_4_^•−^ and •OH, with the resulting HCO_3_^•−^ and CO_3_^•−^ being less potent than SO_4_^•−^ and •OH (Equations (11) and (12)). Additionally, the addition of HCO_3_^−^ renders the solution alkaline, thereby reducing the degradation efficiency of MB.

(8) $${\mathrm{CO}}_{3}^{2-}+\cdot \mathrm{OH}\;\to \;{\cdot \mathrm{CO}}_{3}^{-}+{\mathrm{OH}}^{-}$$

(9) $${\mathrm{CO}}_{3}^{\;2-}+{\mathrm{SO}}_{4}^{\cdot -}\;\to \;{\cdot \mathrm{CO}}_{3}^{-}+{\mathrm{SO}}_{4}^{2-}$$

(10) $${\mathrm{NO}}_{3}^{-}+{\mathrm{OH}}^{\cdot -}\;\to \;{\mathrm{OH}}^{-}+{\mathrm{NO}}_{3}^{\cdot -}$$

(11) $${\mathrm{HCO}}_{3}^{-}+\cdot \mathrm{OH}\;\to \;{\mathrm{CO}}_{3}^{\cdot -}+H_{2}O$$

(12) $${HCO}_{3}^{-}+{\mathrm{SO}}_{4}^{\cdot -}\;\to \;{\mathrm{SO}}_{4}^{-}+{HCO}_{3}^{\cdot -}$$

### 3.3. Reusability and Stability

The reusability and stability of ISPEG were also evaluated. During the first three cycles, the efficiency of MB removal decreased gradually, but after the fourth times use, approximately 82.3% of MB was removed (Figure S3b in Supplementary Materials). The decline in MB removal efficiency is attributed to the inevitable consumption of Fe^0^. Although the MB removal efficiency of ISPEG after five cycles was slightly reduced compared to the fresh material, it still exhibited good catalytic performance. Overall, ISPEG demonstrated satisfactory reusability and long-term stability, indicating potential for practical applications. Besides decolorization, the degree of MB mineralization is crucial for actual industrial applications.

### 3.4. Identification of Reactive Oxygen Species and Degradation Mechanisms

**Figure 4:**
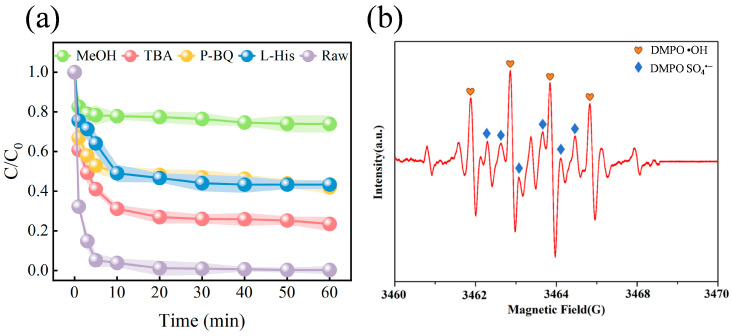
(**a**) Effect of scavengers on MB degradation (**b**) EPR spectra of DMPO- •OH and DMPO- SO_4_^•−^.

The above experimental results showed that ISPEG could effectively activate PDS to improve the degradation of MB. Therefore, it was necessary to investigate the activation mechanism in the ISPEG/PDS system. Bursting experiments were carried out using tert-butanol (TBA) and methanol (MeOH) as radical scavengers, where TBA was the scavenger of •OH with a rate constant of 4.8–7.6 × 108 M^−1^s^−1^ and MeOH as the scavenger of SO_4_^•−^ and •OH bursting agents (1.6–7.7 × 107 M^−1^s^−1^ and 1.2–2.8 × 109 M^−1^s^−1^) [43,44]. P-benzoquinone (p-BQ) was the scavenger of O_2_^•−^ (0.9–1.0 × 109 M^−1^s^−1^) and histidine (L-His) was the scavenger of ^1^O_2_ [45]. Bursting experiments were performed in the presence of various scavengers and the results were shown in Figure 4a. The inhibitory effect of MeOH on MB degradation was greater than that of TBA and p-BQ, with MB removals of 24.6%, 78.3%, and 68.8% after 60 min. The addition of L-His reduced MB removal to 93.9%, which implies that the ^1^O_2_ contribution was negligible. Therefore, it could be surmised that SO_4_^•−^, •OH, and O_2_^•−^ were all involved in the degradation process, with SO_4_^•−^ being the dominant ROS. EPR test was performed to further elucidate accurately the presence of ROS in the ISPEG/PDS system (Figure 4b and Figure S4a in Supplementary Materials). Typical signal peaks of DMPO- SO_4_^•−^, DMPO- •OH and DMPO- O_2_^•−^ could be observed, implying that the system produced SO_4_^•−^, •OH and O_2_^•−^, which is consistent with the free radical burst results [46]. To understand the source of O_2_^•−^, we blew nitrogen and oxygen into the solution to examine the degradation rate of MB (Figure S4b in Supplementary Materials). Compared to oxygen, the MB degradation rate decreased by 21.6% after the passage of nitrogen. Combined with the inhibition of MB by the addition of p-BQ, this suggested that the dissolved oxygen was mainly attributed to the dissolved oxygen in solution, and the O_2_^•−^ came from the reaction between the catalyst and the substances in the system.

**Figure 5:**
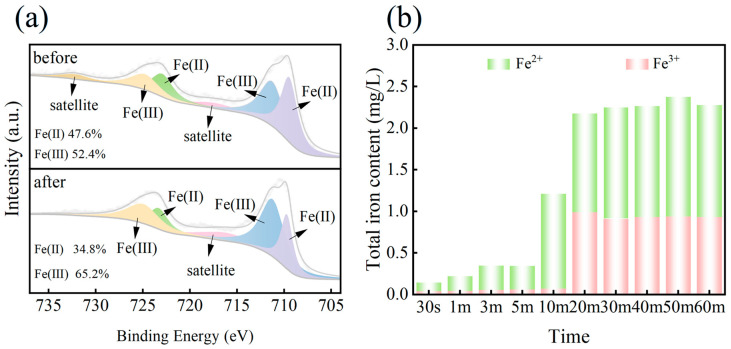
(**a**) XPS profiles of Fe 2p before and after degradation, (**b**) process changes in Fe^2+^/Fe^3+^.

To figure out how free radicals are produced in our reaction system, the XPS spectra of ISPEG before and after the reaction were investigated. As shown in Figure 5a, the Fe 2p 3/2 peak split into two peaks at 710.5 eV and 712.5 eV, representing Fe(II) and Fe(III), respectively. The Fe 2p 1/2 peak also splits into two peaks at 724 eV (Fe(II)) and 726 eV (Fe(III)). The peaks at 717.2 eV and 733.4 eV were satellite peaks. The peak of Fe(II) decreased from 47.6% to 34.8% and Fe(III) increased from 52.4% to 65.2% after the reaction (Table S3 in Supplementary Materials). This was due to the generation of Fe(III) due to the typical reaction between Fe(II) and PDS. This electron transfer between Fe(II) and Fe(III) accelerated the decomposition of PDS and maintains a high catalytic activity. The leaching of iron ions during the activation of PDS was also tested (Figure 5b), and the results indicated that the leaching concentrations of Fe^2+^ and Fe^3+^ were very low in the first 10 min, implying that the early process of degrading MB was dominated by O_2_^•−^, and the later process with the increase in Fe^2+^ with the decrease in PDS was SO_4_^•−^ dominated.

**Figure 6:**
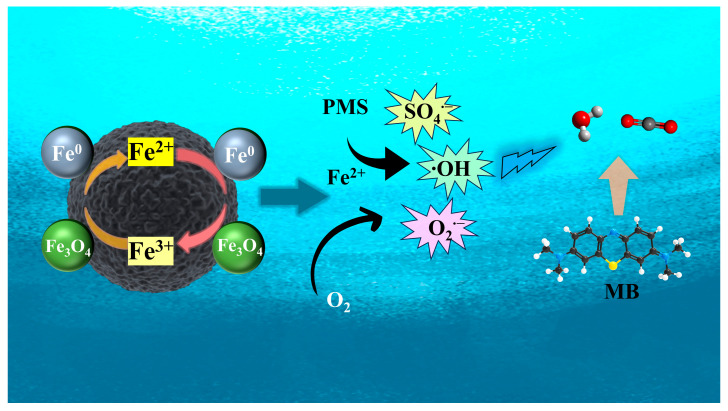
Schematic illustration of MB removal mechanism.

Based on the above analysis, a possible reaction mechanism for pollutant degradation in the ISPEG/PDS system was proposed (Figure 6). In ISPEG30, Fe^0^ acts as a reaction center directly reacting with PDS to generate Fe^2+^. The generated Fe^2+^ further activated PDS to generate SO_4_^•−^. Then, SO_4_^•−^ reacted with MB on the surface of ISPEG30, resulting in a sharp decrease in concentration. In addition, part of the generated SO_4_^•−^ could be converted to •OH, which is further involved in MB degradation. Fe^2+^ also reacted with oxygen to generate O_2_^•−^, which was another major factor in MB degradation. In addition, Fe_3_O_4_ leached Fe^2+^ and Fe^3+^ to participate in the reaction, where Fe^3+^ could be further reduced to Fe^2+^ by Fe^0^, repeating the above cycle, which was a specific manifestation of the synergistic effect of Fe^0^ and Fe_3_O_4_. The C=O group on the ISPEG surface may activate PDS to generate O_2_^•−^, and the subsequent decrease in C=O group content on the ISPEG surface may serve as evidence of their involvement [47]. Overall, free radicals (SO_4_^•−^, •OH, and O_2_^•−^) were all associated with the degradation of MB in the ISPEG/PDS system, with SO_4_^•−^ probably playing a dominant role.

(13) $${\mathrm{Fe}}^{0}+S_{2}O_{8}^{2-}\to {\mathrm{Fe}}^{2+}+2{\mathrm{SO}}_{4}^{2-}$$

(14) $${\mathrm{Fe}}^{2+}+S_{2}O_{8}^{2-}\to {\mathrm{SO}}_{4}^{\cdot -}+{\mathrm{SO}}_{4}^{2-}+{\mathrm{Fe}}^{3+}$$

(15) $${\mathrm{SO}}_{4}^{\cdot -}+H_{2}O\to {\mathrm{SO}}_{4}^{2-}+\cdot \mathrm{OH}+H_{2}O$$

(16) $${\mathrm{Fe}}^{2+}+O_{2}\to O_{2}^{\cdot -}+{\mathrm{Fe}}^{3+}$$

(17) $${2\mathrm{Fe}}^{3+}+{\mathrm{Fe}}^{0}\to {3\mathrm{Fe}}^{2+}$$

(18) $${\mathrm{Fe}}^{3+}+H_{2}O\to {\mathrm{Fe}}^{2+}+\cdot \mathrm{OH}+H^{+}$$

### 3.5. Degradation Pathway of MB

**Figure 7:**
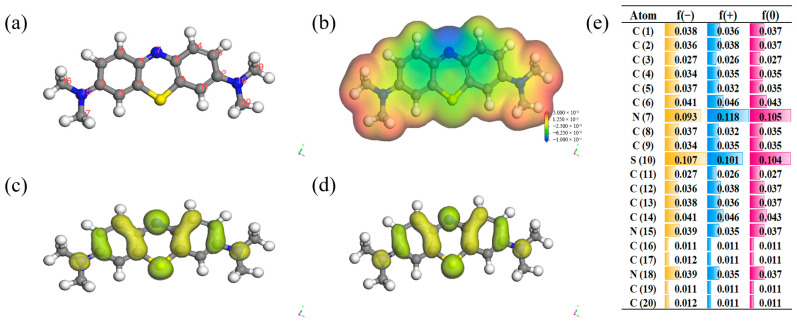
Chemical structure of MB (**a**) and molecular electrostatic potential (ESP) (**b**); HOMO and LUMO distributions (**c**,**d**); calculated Fukui index (**e**).

Frontier orbital theory is widely employed for the prediction of reaction mechanisms and determination of pathways for electron transfer. The MB structure was first geometrically optimized through the B3LYP basis group and solvation model in the DMo13 module, as shown in Figure 7a. The highest occupied orbital (HOMO) of the MB molecule suggests enhanced electron escape ability and susceptibility to electrophilic attack by •OH and SO_4_^•−^; conversely, the lowest occupied orbital (LUMO) of the MB molecule indicates reduced electron escape ability and resistance to electrophilic attack by •OH and SO_4_^•−^. The figure clearly illustrates that the benzene ring attached to the 7N and 10S atoms serves as the primary location for both HOMO and LUMO, indicating its significance as the main reaction site for electron gain and loss (Figure 7c,d). The electrostatic potential distribution on the van der Waals surface of the MB molecule is depicted in Figure 7b, wherein atoms exhibiting a negative ESP denote electron-rich regions, while those with a positive ESP indicate electron-deficient regions. The Fukui function not only predicts reaction sites but also determines the likelihood of electrophilic and nucleophilic reactions, encompassing anodic oxidation as well as susceptibility to attack by reactive species such as •OH and SO_4_^•−^. The highest f (−) value is observed for the 7N and 10S atoms in Figure 7e, indicating its susceptibility to attack by electrophilic species such as •OH and SO_4_^•−^.

**Figure 8:**
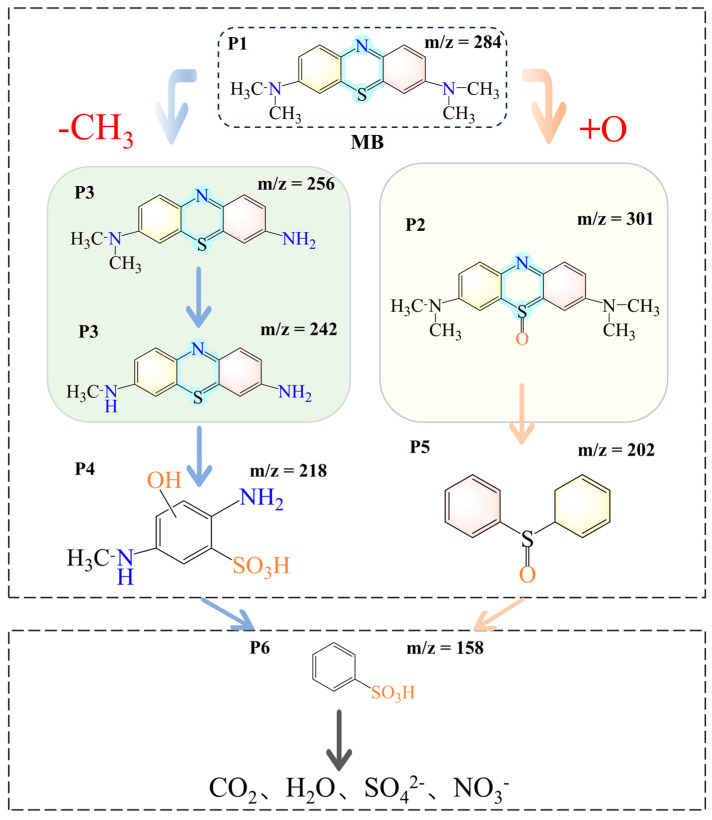
Degradation pathway of MB.

The intermediates generated by the degradation of MB by ISPEG30 were characterized by liquid chromatography (LC-MS), and the degradation pathway of MB was proposed (Figure S5). As shown in Figure 8, the degradation of MB was mainly attributed to demethylation and hydroxylation processes. In the first pathway the N-C bond between the methyl group and the N atom of MB was easily broken, leading to the substitution of the methyl group by H to form a new product, corresponding to the loss of 2 methyl groups (*m*/*z* 256) and 3 methyl groups (*m*/*z* 242) of MB [48,49]. On the other hand, oxidation of the C-S+=C group on the thionine ring forms the m/z 301 intermediate. At this point, the O atom bonds to sulfur (S=O), and the H atom bonds to the N atom (N-H). The oxidation of 3,7-bis(dimethylamine)-10H-phenothiazine 5-oxide (*m*/*z* 301) then continued to give diphenyl sulfoxide (*m*/*z* 202), which is consistent with the results described in the literature. Finally, the intermediate could be further decomposed into simple inorganic molecules, such as carbon dioxide, water, sulfate radicals, and nitrate radicals. The mineralization degree of TCH was determined through TOC testing. After 60 min, the removal rate of TOC in TCH solution was 76.9%, which indicated that the catalyst had good mineralization ability and confirming that the organic pollutants had been mineralized into CO_2_, H_2_O and inorganic ions.

### 3.6. MB Degradation in Actual Water

**Figure 9:**
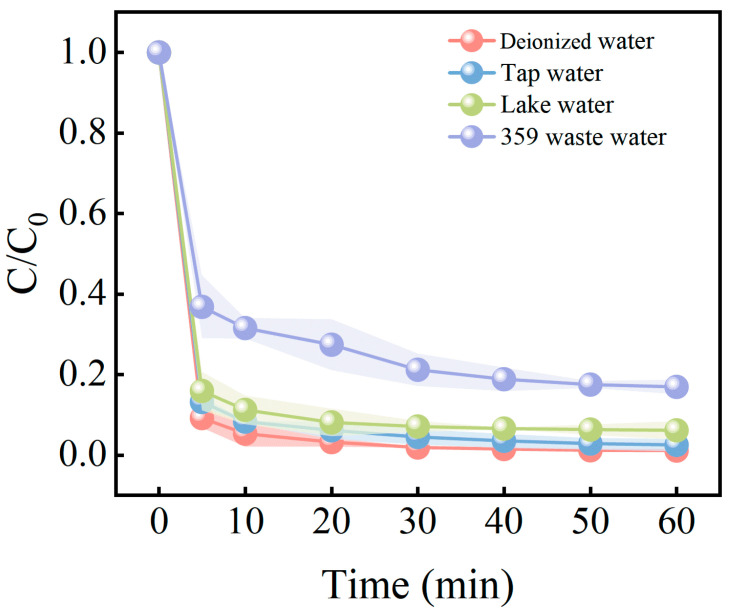
Different water matrixes on MB degradation in ISPEG/PDS system.

The practicality of the catalyst is a key factor in assessing its application potential. In order to further investigate the performance of the ISPEG/PDS system in practical applications, three typical real water samples were selected for testing. The obtained water samples were first filtered out of impurities through a 0.45 μm aqueous filter membrane and their water quality parameters were determined, and the water quality parameters of the typical water samples are shown in Table 1. The degradation rates and reaction rate constants of the actual water samples are shown in Figure 9. Using ultrapure water as the control sample group, the degradation rate of MB in 359 dye wastewater was 83.1% within 60 min. This may be due to the fact that the organic content of 359 dye wastewater is much higher than the aqueous matrix of ultrapure water, which will consume more reactive radicals. The degradation efficiencies of MB in municipal water supply and Tiger Ice Pond water were 97.5% and 93.9%, respectively, which were very similar to that in deionized water (98.9%), suggesting that the impact on the system was minimal. These results indicate that the ISPEG/PDS system is suitable for practical wastewater treatment applications.

## 4. Conclusions

In this study, an ISPEG30 magnetic catalyst was successfully prepared with excellent redox properties and magnetic properties. The prepared magnetic catalyst was able to effectively activate persulfate and achieve 99.7% MB removal within 60 min under suitable operating parameter conditions (ISPEG30 = 0.2 g/L, PDS = 1.2 mM, unadjusted pH). The removal of MB could reach more than 80% after 5 consecutive cycles. In addition, from the mechanistic study, we found that SO_4_^•−^ plays a dominant role and •OH and O_2_^•−^ play a secondary role in the degradation of MB in the ISPEG/PDS system. Meanwhile, the key role of Fe^0^ and Fe_3_O_4_ in promoting Fe^2+^/Fe^3+^ cycling in PDS activation was elucidated. In addition, we identified the degradation pathways of MB by intermediate LC-MS analysis, which were mainly demethylation and hydroxylation reactions. The catalyst has a wide pH range, excellent reusability, and practicality, and provides a new idea for developing functionalized use of various wastes.

## Figures and Tables

**Table 1:** Some physicochemical properties.

Water Sources	CODcr (mg/L)	pH	Conductivity (μS/cm)	MB Concentration (mg/L)
Lake water	41	7.25	187	Not detected
Tap water	4.53	7.12	191	Not detected
359 dye wastewater	5845	10.1	17,209	20

## Data Availability

All data generated or analyzed during this study are included in this published article and its Supplementary Information Files.

## Supplementary Materials

The following supporting information can be downloaded at: https://www.mdpi.com/article/10.3390/nano15201585/s1, Text S1: Methods of LC-MS. S1; Figure S1: EDS and elemental percentages; Figure S2: FTIR at different temperatures; Figure S3: (a) Magnetic hysteresis loops (the inset shows the magnetic responsive performance) for ISPE30, (b) Five cycle experiment of ISPE30; Figure S4: (a) EPR spectra of DMPO-O_2_^•−^, (b) MB removal under N_2_ and O_2_ conditions; Figure S5: Mass spectrometry of intermediates of MB; Table S1: Components of IS and catalyst feedstock after co-pyrolysis detected by XRF; Table S2: The percentage of Fe (II) and Fe (III) of ISPE before and after utilization; Table S3: Parameters related to kinetic analysis curves.

## References

[1] Wang C.Q., Huang R., Sun R.R., Yang J.P., Sillanpää M. (2021). A review on persulfates activation by functional biochar for organic contaminants removal: Synthesis, characterizations, radical determination, and mechanism. J. Environ. Chem. Eng..

[2] Kang Z.Y., Jia X.G., Zhang Y.C., Kang X.X., Ge M., Liu D., Wang C., He Z. (2022). A Review on Application of Biochar in the Removal of Pharmaceutical Pollutants through Adsorption and Persulfate-Based AOPs. Sustainability.

[3] Rodríguez-Chueca J., García-Cañibano C., Lepistö R.J., Encinas Á., Pellinen J., Marugán J. (2019). Intensification of UV-C tertiary treatment: Disinfection and removal of micropollutants by sulfate radical based Advanced Oxidation Processes. J. Hazard. Mater..

[4] Ding C., Xiao S., Lin Y., Yu P., Zhong M.-e., Yang L., Wang H., Su L., Liao C., Zhou Y. (2019). Attapulgite-supported nano-Fe0/peroxymonsulfate for quinclorac removal: Performance, mechanism and degradation pathway. Chem. Eng. J..

[5] Li D.H., Zhang S.X., Li S.N., Tang J.C., Hua T., Li F. (2023). Mechanism of the application of single-atom catalyst-activated PMS/PDS to the degradation of organic pollutants in water environment: A review. J. Clean. Prod..

[6] Huang W., Xiao S., Zhong H., Yan M., Yang X. (2021). Activation of persulfates by carbonaceous materials: A review. Chem. Eng. J..

[7] Lu J., Lu Q., Di L., Zhou Y., Zhou Y. (2023). Iron-based biochar as efficient persulfate activation catalyst for emerging pollutants removal: A review. Chin. Chem. Lett..

[8] Zhang X.-W., Wang F., Wang C.-C., Wang P., Fu H., Zhao C. (2021). Photocatalysis activation of peroxodisulfate over the supported Fe_3_O_4_ catalyst derived from MIL-88A(Fe) for efficient tetracycline hydrochloride degradation. Chem. Eng. J..

[9] Li X., Rykov A.I., Zhang B., Zhang Y., Wang J. (2016). Graphene encapsulated Fe x Co y nanocages derived from metal–organic frameworks as efficient activators for peroxymonosulfate. Catal. Sci. Technol..

[10] He L., Wu D., Rong H., Li M., Tong M., Kim H. (2018). Influence of Nano- and Microplastic Particles on the Transport and Deposition Behaviors of Bacteria in Quartz Sand. Environ. Sci. Technol..

[11] Zhang K.-X., Song C., Zhao S., Yan Z., Feng L.-J., Wang S.-G. (2021). AOPs enhance the migration of polystyrene nanoparticles in saturated quartz sand. Environ. Sci. Process. Impacts.

[12] Song H., Tsang D.C., Kwon G., Kwon E.E., Cho D.-W. (2019). Coupling carbon dioxide and magnetite for the enhanced thermolysis of polyvinyl chloride. Sci. Total Environ..

[13] Kwon G., Cho D.-W., Wang H., Bhatnagar A., Song H. (2020). Valorization of plastics and paper mill sludge into carbon composite and its catalytic performance for acarbon material consisted of the multi-layerzo dye oxidation. J. Hazard. Mater..

[14] Ma D., Yang Y., Liu B., Xie G., Chen C., Ren N., Xing D. (2021). Zero-valent iron and biochar composite with high specific surface area via K_2_FeO_4_ fabrication enhances sulfadiazine removal by persulfate activation. Chem. Eng. J..

[15] Großwendt F., Bürk V., Kopanka B., Jäger S., Pollak S., Leich L., Röttger A., Petermann M., Weber S. (2023). A novel powder-metallurgical eco-friendly recycling process for tool steel grinding sludge. J. Clean. Prod..

[16] Guo Y., Zhang Z., Shi W., Zhang B., Li W., Cui F., Lens P.N.L. (2021). Evolution of the sludge mineral composition enhances operation performance of the aerobic granular sludge reactor coupled with iron electrolysis. J. Clean. Prod..

[17] Yao Z., Seong H.J., Jang Y.-S. (2022). Environmental toxicity and decomposition of polyethylene. Ecotoxicol. Environ. Saf..

[18] Jin H.-Y., He Z.-W., Ren Y.-X., Zou Z.-S., Tang C.-C., Zhou A.-J., Liu W., Li Z., Wang A. (2024). Revealing the roles of biochar derived from iron-rich fermented sludge residue in anaerobic digestion. Chem. Eng. J..

[19] Zhang Z., Du C., Zhang Y., Yu G., Xiong Y., Zhou L., Liu Y., Chi T., Wang G., Su Y. (2022). Degradation of oxytetracycline by magnetic MOFs heterojunction photocatalyst with persulfate: High stability and wide range. Environ. Sci. Pollut. Res..

[20] Zhao Y.S., Sun C., Sun J.Q., Zhou R. (2015). Kinetic modeling and efficiency of sulfate radical-based oxidation to remove p-nitroaniline from wastewater by persulfate/Fe_3_O_4_ nanoparticles process. Sep. Purif. Technol..

[21] Criveanu A., Dumitrache F., Fleaca C., Gavrila-Florescu L., Lungu I., Morjan I.P., Socoliuc V., Prodan G. (2023). Chitosan-coated iron oxide nanoparticles obtained by laser pyrolysis. Appl. Surf. Sci. Adv..

[22] Meng F., Song M., Song B., Wei Y., Cao Q., Cao Y. (2020). Enhanced degradation of Rhodamine B via α-Fe2O3 microspheres induced persulfate to generate reactive oxidizing species. Chemosphere.

[23] Pu M., Wan J., Zhang F., Brusseau M.L., Ye D., Niu J. (2021). Insight into degradation mechanism of sulfamethoxazole by metal-organic framework derived novel magnetic Fe@C composite activated persulfate. J. Hazard. Mater..

[24] Sun X.M., Li Y. (2004). Colloidal carbon spheres and their core/shell structures with noble-metal nanoparticles. Angew. Chem..

[25] Yu J., Tang L., Pang Y., Zeng G., Feng H., Zou J., Wang J., Feng C., Zhu X., Ouyang X. (2020). Hierarchical porous biochar from shrimp shell for persulfate activation: A two-electron transfer path and key impact factors. Appl. Catal. B Environ..

[26] Lu L., Ai Z., Li J., Zheng Z., Li Q., Zhang L. (2007). Synthesis and Characterization of Fe−Fe_2_O_3_ Core−Shell Nanowires and Nanonecklaces. Cryst. Growth Des..

[27] Ai Z.H., Gao Z.T., Zhang L.Z., He W.W., Yin J.J. (2013). Core–shell structure dependent reactivity of Fe@ Fe_2_O_3_ nanowires on aerobic degradation of 4-chlorophenol. Environ. Sci. Technol..

[28] Jiang B., Zhang Y.S., Li C., Guo J.Q., Sun C.M. (2023). Zero-valent iron loaded on N-doped biochar fabricated by one-step pyrolysis of K_2_FeO_4_ and coffee grounds as a persulfate activator for Bisphenol A degradation. Process Saf. Environ. Prot..

[29] Qin J.H., Wang X., Deng M.J., Li H.S., Lin C.X. (2022). Red mud-biochar composites (co-pyrolyzed red mud-plant materials): Characteristics and improved efficacy on the treatment of acidic mine water and trace element-contaminated soils. Sci. Total Environ..

[30] Guo Z., Bai G., Huang B., Cai N., Guo P., Chen L. (2021). Preparation and application of a novel biochar-supported red mud catalyst: Active sites and catalytic mechanism. J. Hazard. Mater..

[31] Chen Y., Su R., Xu F., Ma M., Wang Y., Ma D., Li Q. (2024). Oxygen-containing functional groups in Fe_3_O_4_@three-dimensional graphene nanocomposites for enhancing H_2_O_2_ production and orientation to _1_O_2_ in electro-Fenton. J. Hazard. Mater..

[32] Sun H., Zhou G., Liu S., Ang H.M., Tadé M.O., Wang S. (2012). Nano-Fe_0_ Encapsulated in Microcarbon Spheres: Synthesis, Characterization, and Environmental Applications. ACS Appl. Mater. Interfaces.

[33] Fan X., Lin Q., Zheng J., Fu H., Xu K., Liu Y., Ma Y., He J. (2022). Peroxydisulfate activation by nano zero-valent iron graphitized carbon materials for ciprofloxacin removal: Effects and mechanism. J. Hazard. Mater..

[34] Jonidi Jafari A., Kakavandi B., Jaafarzadeh N., Rezaei Kalantary R., Ahmadi M., Akbar Babaei A. (2017). Fenton-like catalytic oxidation of tetracycline by AC@Fe_3_O_4_ as a heterogeneous persulfate activator: Adsorption and degradation studies. J. Ind. Eng. Chem..

[35] Saidani A., Boudraa R., Fendi K., Benouadah L., Benabbas A., Djermoune A., Salvestrini S., Bollinger J.-C., Alayyaf A.A., Mouni L. (2025). Effect of Calcination Temperature on the Photocatalytic Activity of Precipitated ZnO Nanoparticles for the Degradation of Rhodamine B Under Different Light Sources. Water.

[36] Wu X., Li T., Wang R., Zhang Y., Liu W., Yuan L. (2021). One-pot green synthesis of Zero-Valent iron particles supported on N-Doped porous carbon for efficient removal of organic pollutants via Persulfate Activation: Low iron leaching and degradation mechanism. Sep. Purif. Technol..

[37] Guo M., Han Y., Gao H., Xu Z., Wang D., Peng Z., Hou H. (2024). Red mud-based biochar activated peroxydisulfate under visible light for tetracycline removal: Synergistic effect of FeAl_2_O_4_ photocatalysis. Sep. Purif. Technol..

[38] Li X., Zhang W., Liu Z., Wang S., Zhang X., Xu B., Yu P., Xu Y., Sun Y. (2022). Effective removal of tetracycline from water by catalytic peroxymonosulfate oxidation over Co@MoS_2_: Catalytic performance and degradation mechanism. Sep. Purif. Technol..

[39] Zheng M.M., Li Y.H., Cao M.H., Guo Y., Qiu G., Tu S., Xiong S., Fang D. (2024). Amino acid promoted oxidation of atrazine by Fe_3_O_4_/persulfate. Heliyon.

[40] Jin Z.A., Zhao X.T., Zhang M., Li Y., Guo J., Lan Y., Chen C. (2024). Waste self-heating bag derived CoFe_2_O_4_ composite enhances peroxymonosulfate activation: Performance, mechanism, and adaptability under high-salinity conditions. J. Water Process Eng..

[41] Chen Y., Cui K., Cui M., Liu T., Chen X., Chen Y., Nie X., Xu Z., Li C.-X. (2022). Insight into the degradation of tetracycline hydrochloride by non-radical-dominated peroxymonosulfate activation with hollow shell-core Co@NC: Role of cobalt species. Sep. Purif. Technol..

[42] Li B., Li C.-X., Wang Y., Xu W., Cui K., Zhan X., Deng R., Zhang X. (2023). In-situ preparation of yeast-supported Fe0@Fe_2_O_3_ as peroxymonosulfate activator for enhanced degradation of tetracycline hydrochloride. Chemosphere.

[43] Huang Q.L., Chen C.J., Zhao X.L., Bu X.Y., Liao X.F., Fan H., Gao W., Hu H., Zhang Y., Huang Z. (2021). Malachite green degradation by persulfate activation with CuFe_2_O_4_@biochar composite: Efficiency, stability and mechanism. J. Environ. Chem. Eng..

[44] Zhu S., Huang X., Ma F., Wang L., Duan X., Wang S. (2018). Catalytic Removal of Aqueous Contaminants on N-Doped Graphitic Biochars: Inherent Roles of Adsorption and Nonradical Mechanisms. Environ. Sci. Technol..

[45] Liao L., Yue H., Cui Y. (2011). Crosslink Polymerization Kinetics and Mechanism of Hydrogels Composed of Acrylic Acid and 2-Acrylamido-2-methylpropane Sulfonic Acid. Chin. J. Chem. Eng..

[46] Tian L., Liu S.-S., Jiang X.-H., Chen L.-S., Wu S.-L., Xiao W.-J., Fan J.-P., Wu D.-S., Zou J.-P. (2021). Selective oxidation of diclofenac sodium with different electronegative moieties via coexisting SO_4_^−^ and OH. Sci. Total Environ..

[47] Zhuang Y., Liu J., Yuan S., Ge B., Du H., Qu C., Chen H., Wu C., Li W., Zhang Y. (2020). Degradation of octane using an efficient and stable core-shell Fe_3_O_4_@C during Fenton processes: Enhanced mass transfer, adsorption and catalysis. Appl. Surf. Sci..

[48] Trandafilović L.V., Jovanović D.J., Zhang X., Ptasińska S., Dramićanin M.D. (2017). Enhanced photocatalytic degradation of methylene blue and methyl orange by ZnO:Eu nanoparticles. Appl. Catal. B Environ..

[49] Houas A., Lachheb H., Ksibi M., Elaloui E., Guillard C., Herrmann J.-M. (2001). Photocatalytic degradation pathway of methylene blue in water. Appl. Catal. B Environ..

