# Supplementary material for: Peroxydisulfate Activation by Pyrolysis Products of Iron Grinding Sludge and Polyethylene Glycol for Methylene Blue Degradation: Mechanism and Performance
Source: Nanomaterials (Basel). 2025 Oct 17;15(20):1585. doi: 10.3390/nano15201585 (PMC12567454; doi:10.3390/nano15201585)
Supplement: Supplementary file 1 [file nanomaterials-15-01585-s001.zip › nanomaterials-3868678-supplementary.pdf]

nanomaterials

Supplementary Materials

Peroxydisulfate Activation by Pyrolysis Products of Iron Grinding Sludge and Polyethylene Glycol for Methylene Blue Degradation: Mechanism and Performance

De-Feng Kong [1,2,†], Hui-Lai Liu [1,†], Yi Han [3], Ting Shi [3], De-Jin Wang [3,*] and Xing Chen [1,*]

[1] School of Resources and Environmental Engineering, Hefei University of Technology, Hefei 230009, China; kdf_001.student@sina.com (D.-F.K.); 2024820023@hfut.edu.cn (H.-L.L.)
[2] Anhui Haoyue Ecological Technology Co., Ltd., Hefei 230009, China
[3] College of Resources and Environment, Anqing Normal University, Anqing 246011, China; yihan@whu.edu.cn (Y.H.); 18324988735@163.com (T.S.)
* Correspondence: wangdj@aqnu.edu.cn (D.-J.W.); xingchen@hfut.edu.cn (X.C.)
† These authors contributed equally to this work.

Academic Editor(s): Name

Received: 27 August 2025
Revised: 3 October 2025
Accepted: 13 October 2025
Published: 17 October 2025

Citation: Kong, D.-F.; Liu, H.-L.; Han, Y.; Shi, T.; Wang, D.-J.; Chen, X. Peroxydisulfate Activation by Pyrolysis Products of Iron Grinding Sludge and Polyethylene Glycol for Methylene Blue Degradation: Mechanism and Performance. *Nanomaterials* **2025**, *15*, 1585. https://doi.org/10.3390/nano15201585

Copyright: © 2025 by the authors. Licensee MDPI, Basel, Switzerland. This article is an open access article distributed under the terms and conditions of the Creative Commons Attribution (CC BY) license (https://creativecommons.org/licenses/by/4.0/).

Text S1. Methods of LC-MS.

The mobile phase consisting of 0.1% formic acid in water (A) and acetonitrile (B) at 0.1 mL/min. The acetonitrile volume ratio increased from 20% to 80% by a linear gradient progress in 20 min. The injection volume was 1 μL. The MS spectrum was recorded in the m/z range of 50–500 in positive mode.

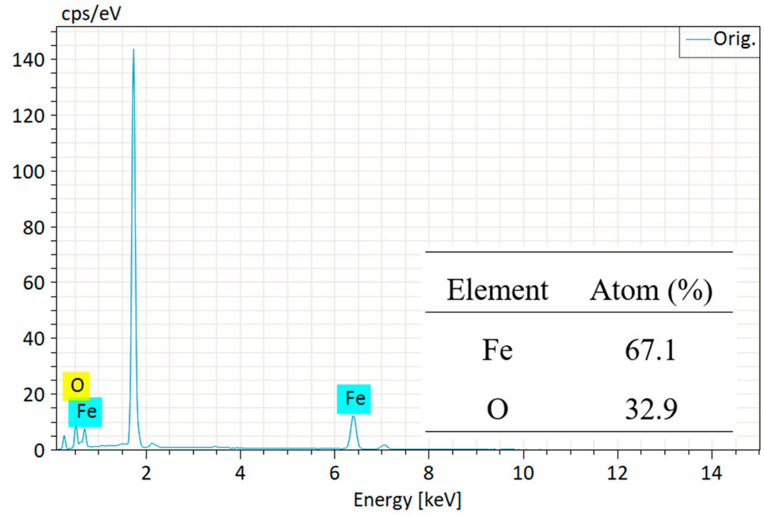

Figure S1. EDS and elemental percentages.

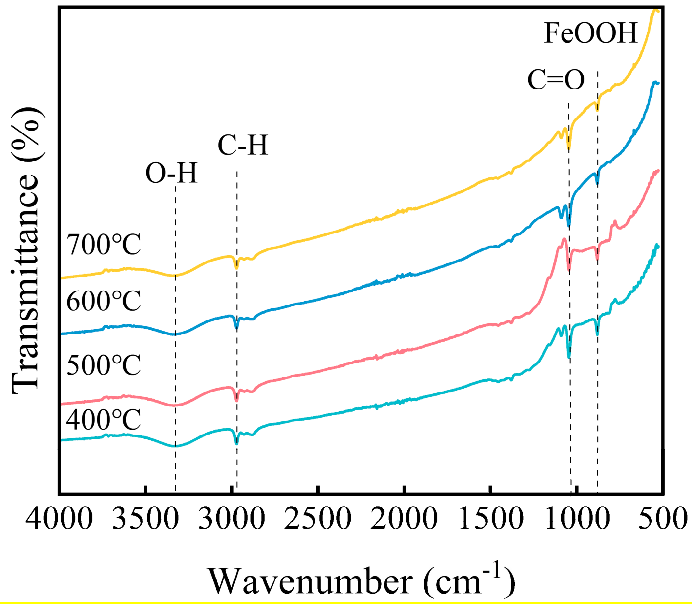

Figure S2. FTIR at different temperatures.

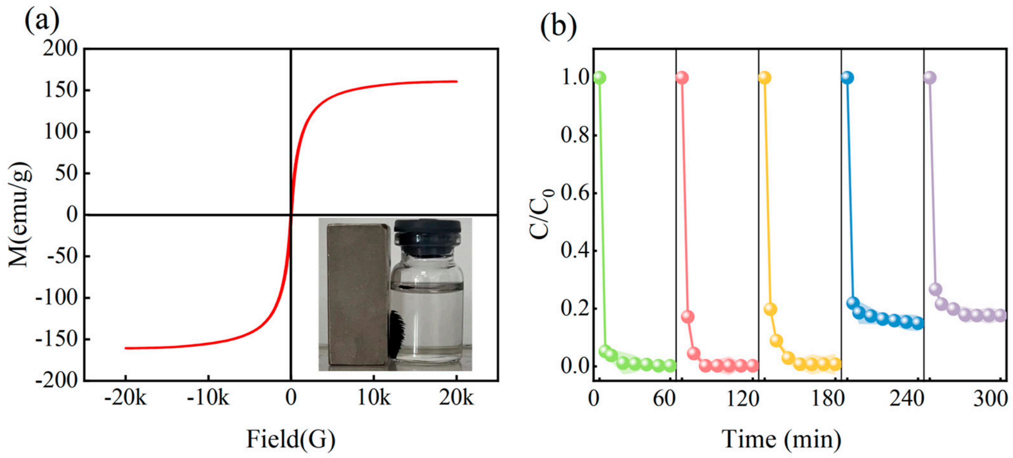

Figure S3. (**a**) Magnetic hysteresis loops (the inset shows the magnetic responsive performance) for ISPE30, (**b**) Five cycle experiment of ISPE30.

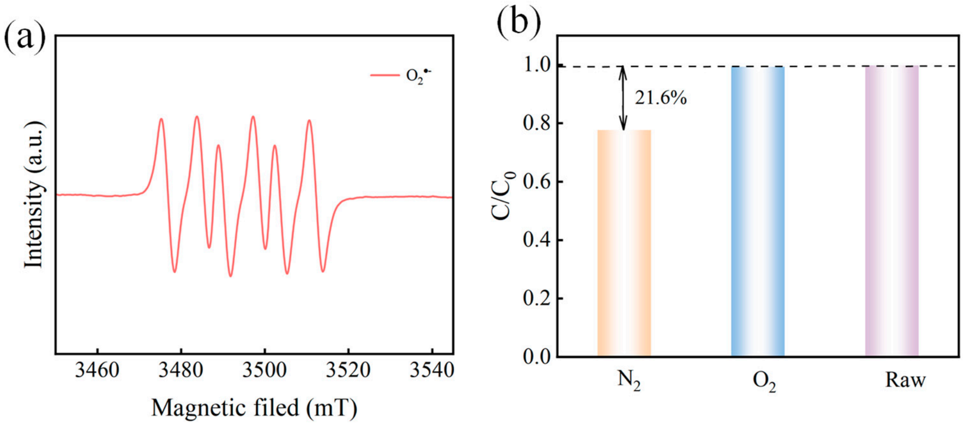

Figure S4. (a) EPR spectra of DMPO-$O_2^{\bullet-}$, (b)MB removal under N_2 and O_2 conditions.

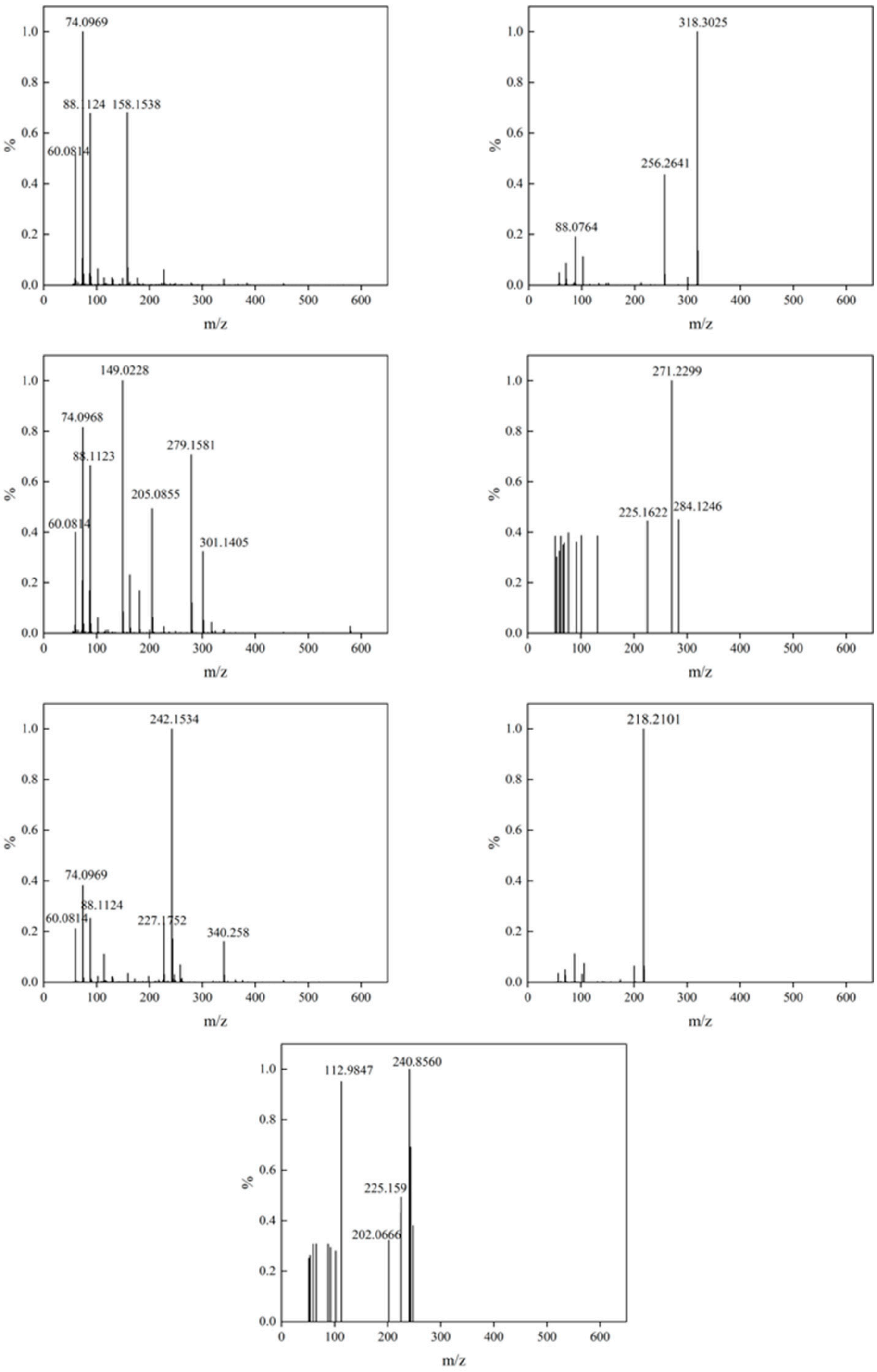

Figure S5. Mass spectrometry of intermediates of MB.

Table S1. Components of IS and catalyst feedstock after co-pyrolysis detected by XRF.

Component	Al	Si	Mn	Fe	Cu	Na	S	Ca
IS	0.419	2.504	0.349	95.604	0.608	0.164	0.110	0.051
ISPE70	0.408	12.893	0.277	85.074	0.475	0.195	0.087	0.051

Table S2. Parameters related to kinetic analysis curves.

		R^2	K_{obs}
IS/PE ratio	ISPE10	0.96	0.14
	ISPE30	0.95	0.17
	ISPE50	0.95	0.16
	ISPE70	0.96	0.16
	ISPE90	0.97	0.15
pyrolysis temperatures	400 °C	0.85	0.13
	500 °C	0.93	0.16
	600 °C	0.95	0.17
	700 °C	0.78	0.12
ISPE30 dosage	0.1 g/L	0.98	0.14
	0.2 g/L	0.85	0.17
	0.4 g/L	0.88	0.16
	0.6 g/L	0.94	0.18
	0.8 g/L	0.95	0.17
PDS concentration	0 mM	0.99	0.01
	0.4 mM	0.95	0.17
	0.8 mM	0.93	0.29
	1.2 mM	0.90	0.32
	1.6 mM	0.90	0.30
pH	pH = 3	0.84	0.27
	pH = 5	0.87	0.25
	pH = 7	0.84	0.32
	pH = 9	0.84	0.11
	pH = 11	0.84	0.11
	raw	0.90	0.32
anions	CO_3^{2-}	0.83	0.14
	SO_4^{2-}	0.79	0.12
	HCO_3^-	0.99	0.20
	raw	0.90	0.32

Table S3. The percentage of Fe (II) and Fe (III) of ISPE before and after utilization.

	Before utilization (%)	After utilization (%)
Fe (II)	47.6	52.4
Fe (III)	34.8	65.2
